# Idiotypic and anti-idiotypic antibodies against polycyclic aromatic hydrocarbon in human blood serum are new biomarkers of lung cancer

**DOI:** 10.18632/oncotarget.27126

**Published:** 2019-08-20

**Authors:** Ivan S. Grebenshchikov, Artem E. Studennikov, Vadim I. Ivanov, Natalia V. Ivanova, Victor A. Titov, Natalja E. Vergbickaya, Valentin A. Ustinov

**Affiliations:** ^1^ Federal State Scientific Institute, Federal Research Centre Coal and Coal Chemistry, Siberian Branch of the Russian Academy of Sciences, Institute of Human Ecology, Kemerovo, 650065, Russia; ^2^ Federal State Educational Institute of Higher Professional Education, Kemerovo State University, Kemerovo, 650043, Russia; ^3^ Regional Oncology Clinic, Kemerovo, 650055, Russia

**Keywords:** polycyclic aromatic hydrocarbon, benzo[a]pyrene, anti-idiotypic antibodies, idiotypic antibodies, lung cancer

## Abstract

Evaluation of epidemiologic risk factor in relation to lung cancer invoked by polycyclic aromatic hydrocarbons has been inconsistent. To address this issue, we conducted a prospective evaluation of new biomarkers for lung cancer classified according levels of idiotypic and anti-idiotypic antibodies against polycyclic aromatic hydrocarbons in human blood serum. The blood serums of 557 lung cancer patients and 227 healthy donors were analysis of these antibodies by ELISA. Collected data were regrouped and analyzed by gender, smoking, and age as predictors of risk lung cancer factors. Also, the data of lung cancer patients were additionally analyzed by stages and types of lung cancer, surgery, and chemotherapy. It was suggested to use ratio of idiotypic and anti-idiotypic antibodies rather than distinguish level each of them separately. The ratio of levels in healthy people was 3.32 times higher than in lung cancer patients. This approach gave more precisely results and great prognostic value. The logistic regression model (AUC = 0.9) and neural networks (AUC = 0.95) were built to compare lung cancer patients and healthy donors by predictors. The ELISA data of 49 people random sampled from the originally ELISA data and ELISA data of 52 coal miners as a group of lung cancer risk were confirmed logistic regression model. So, suggested idiotypic and anti-idiotypic antibodies against polycyclic aromatic hydrocarbons were not only shown difference between healthy donors and lung cancer patients also elicited group of lung cancer risk among healthy people.

## INTRODUCTION

Chemical carcinogens of the environment, in particular benzo[a]pyrene (Bp), are significant factors in the occurrence of cancer in humans [1]. The highly carcinogenic following metabolism to highly reactive epoxide metabolites which bind to DNA and proteins [2, 3], and thus acquire the ability to induce the formation of specific antibodies (Abs). It was assumed that Abs against environment chemical carcinogens modulate their biological properties in the human body and are able to influence the processes of initiation and promotion of malignant transformation of cells [1, 4, 5]. Induction of Abs against polycyclic aromatic hydrocarbons (PAHs) was considered by many authors as one of the immunoprophylactic areas of human cancer [6–13]. In this regard, the immunoassay methods have been developed and are in increasing use to quantify PAHs and their metabolites in human biological fluids [14]; PAHs adducts with macromolecules of the organism [15, 16]; Abs against PAHs and them adducts in human serum blood [17–20]; and experimental animals following various immunization protocols [7, 8, 21, 22].

Abs proteomics technology has the potential to become a fundamental technology in drug discovery for development of novel biomarkers and therapeutic targets. Abs are broad using on molecular alterations in lung cancer that are targets for therapy [23]. Also, Abs are using for lung cancer immunodiagnostic. For example, the measurement of Abs against seven tumor-associated antigens by immunoassay was in the early detection of lung cancer [24]. It was evaluated Abs against nine tumor-associated antigens, including p62, p16, Koc, p53, Cyclin B1, Cyclin E, Survivin, HCC1, and RalA by ELISA as serological markers in lung cancer [25] or four different antigens were present in non-small cell lung cancer cells *in situ* [26]. It was successfully identified oxysterol binding protein like 5 and calumenin as potential biomarkers related to metastasis in lung cancer [27]. The accuracy of a panel of proteins and an autoantibody were validated in a population relevant to lung cancer detection and suggested a benefit to combining clinical features with the biomarker results [28]. Other model for lung cancer diagnosis was built based on the blood biomarkers progastrin-releasing peptide, carcinoembryonic antigen, squamous cell carcinoma antigen, and cytokeratin 19 fragment [29].

Risk factors for the development of lung cancer, such as tobacco smoking along [30–32], gender [33], tobacco smoking and gender [34], age, gender, and smoking status [35], immune alterations [36] have been summarized and integrated into comprehensive models of incidence. Previous studies of risk factor for PAHs status among lung cancer patients have typically considered with air pollutants [37] or tobacco smoking and environmental risk factors at the same time [38]. Many of these studies, however, did not classify lung cancer cases jointly by risk factors: tobacco smoking, age, gender, and working conditions using levels of idiotypic (Ab1) and anti-idiotypic (Ab2) Abs against PAHs in blood serum as lung cancer markers. To further clarify the role of idiotypic and anti-idiotypic Abs against low molecular weight xenobiotics in carcinogenesis it is advisable to investigate those in a group of lung cancer patients to compare with healthy people. Preliminarily we found higher levels Abs against PAHs in lung cancer patients which correlated with Bp immunized mouse [22].

## RESULTS

### The levels of idiotypic (Ab1) and anti-idiotypic (Ab2) antibody against PAHs in blood serum of lung cancer patients compares with healthy donors

We collected of healthy people (*n* = 227) and lung cancer patients blood serums (*n* = 557). The conjugate Bp-BSA and mouse idiotypic scFvs against PAHs (pSh) were immobilized on 96 wells plates to determine of Ab1 and Ab2 against PAHs in human blood serum, respectively.

**Figure 1:**
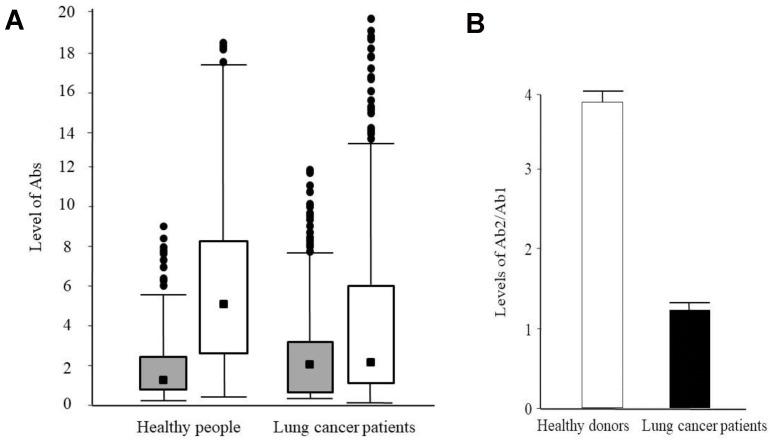
Levels of the idiotypic and anti-idiotypic antibodies against PAHs in blood serum of healthy people and lung cancer patients. (**A**) Box-plots of Ab1 (grey) and Ab2 (white) levels in blood serum of healthy people and lung cancer patients. The scale on the ordinate is levels of Ab1 and Ab2 (see Materials and methods). The values of medians are indicated. (**B**) Ratio of Ab2/Ab1 levels for healthy people (white) and lung cancer patients (black). Values are presented as the means ± S.E.

The Figure 1A shows the analysis of Ab1 and Ab2 detection in the blood serum of healthy persons and lung cancer patients. The methods and criteria of modified Shapiro-Wilk were used to assess the normality distribution of the ELISA data besides the analysis of histograms. The analysis of distribution showed that the data belong to a unimodal distribution with a positive asymmetry, but not a unimodal distribution with a symmetric shape (normal distribution). Ejection points were more than three standard deviations from the median removed by box-plots analysis. Then the data were analyzed by Z adjusted Mann–Whitney *U*-test (Figure 1A). The level of Ab2 (median = 5.25, 2.55:8.41) was 3.95 times higher than Ab1 level (median = 1.33, 0.62:2.68) in healthy people (*p* = 0.001). In lung cancer patients the difference between Ab2 (median = 2.39, 0.94:5.96) and Ab1 levels (median = 2.01, 0.88:3.49) were 1.19 value (*p* = 0.002). The Ab1 levels in lung cancer patients were 1.5 times higher than in healthy individuals (*p =* 0.001). However, the levels of Ab2 in lung cancer patients were 45.5% lower than in healthy people (*p =* 0.001).

The Figure 1B shows the ratio Ab2/Ab1 levels in healthy people and lung cancer patients. The ratio in healthy people (3.95, 1.84:7.51) was 3.32 times higher than in lung cancer patients (1.19, 0.52:2.62). That was why we suggested using the ratio Ab2/Ab1 for lung cancer risk prediction, instead of separately measuring of Ab1 and Ab2 levels separately. This approach would give more precisely results and great prognostic value.

### Breakdown into groups and analysis of groups

**Figure 2:**
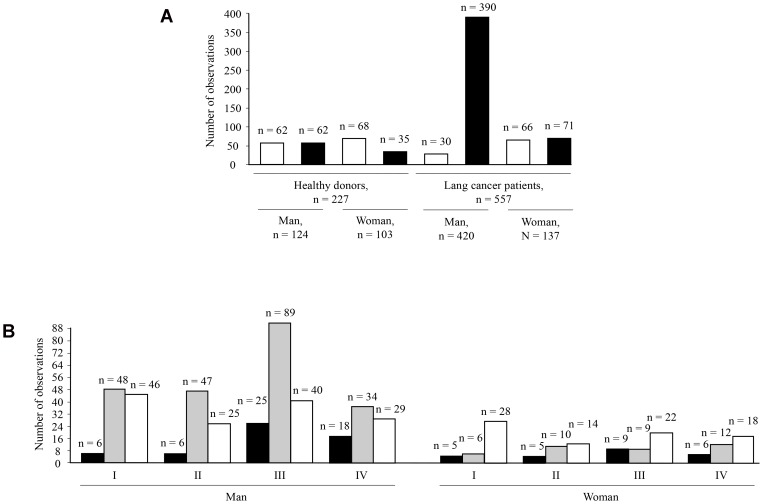
Analysis of amount cases in breakdown groups. The break downing by gender, non-smokers (open bars), and smokers (solid bars) for healthy donors and lung cancer patients (**A**). Analysis of amount cases for lung cancer patients in groups breakdown by gender, lung cancer stages according TNM (I, II, III, and IV), and type of lung cancer: small-cell lung cancer (solid bars), non-small-cell lung cancer (grey bars), and adenocarcinoma (open bars) (**B**).

The ELISA data of healthy donors and lung cancer patients were grouped and broken down with gender and smoking as predictor factors for further analyses (Figure 2A). The analysis of Pearson’s chi-squared test was: χ^2^ = 36.81, *p* = 0.001 for men and women compare with healthy donors and lung cancer patients; χ^2^ = 48.84, *p* = 0.001 for non-smokers and smokers compare with healthy donors and lung cancer patients (data not shown). So, the breakdown of ELISA data by health, gender, and smoking was statistically significant in all cases.

Figure 2B shows distribution of small-cell lung cancer, non-small-cell lung cancer, and adenocarcinoma according to gender and lung cancer stages in studied lung cancer patients’ population. Small-cell lung carcinoma (9.4% of total lung cancer cases) is the rarest type of lung cancer in the Kuzbass region, Russia. Non-small-cell lung cancer was 53% and adenocarcinoma was 38% of the total number of examined cases. The incidence of lung cancer in men (76% of total examined cases) was more common than in women (24% of total examined cases).

The lung cancer patients’ database was supplemented with information on the lung cancer stages (TNM), type of lung cancer, chemotherapy, and surgery. Then, the Kruskal-Wallis test was done to identify correlations and for testing whether samples originate from the same distribution. In the analysis Ab1 and Ab2 were the dependent variables. The lung cancer stages, type of lung cancer, chemotherapy, and surgery were independent variables. So, the test did not reveal any correlations. Based on this, we concluded that the lung cancer stages, type of lung cancer, chemotherapy, and surgery did not affect the levels of Ab1 and Ab2. One of the possible explanation is that the human immune system changes dramatically after the development of lung cancer. Therefore, in lung cancer patients the levels of Ab1 and Ab2 are not a criterion for assessing the development and progression of this disease.

The nonparametric Mann–Whitney *U*-test was applied for data analysis between groups, because of ELISA data belonged to unimodal distribution with a positive asymmetry. Table 1 shows medians values for Ab1, Ab2, and Ab2 divided by Ab1 (Ab2/Ab1) levels and relationship between groups by Z adjusted Mann–Whitney *U*-test.

**Table 1:** ELISA data analysis of healthy donors and lung cancer patients’ based on Ab1 (A), Ab2 (B), and Ab2/Ab1 (C) levels using health, gender and smoking as predictors

A			
Groups	Healthy people, median value of Ab1 (P25:P75)	Lung cancer patients, median value of Ab1 (P25:P75)	Mann–Whitney U-test for data analysis between groups of healthy people and lung cancer patients, *p* values
1. Men total	2.1 (0.95:3.74)	2.09 (0.96:3.78)	> 0.05
1.1. Men non-smokers	2.13 (1.19:3.67)	2.04 (0.97:3.86)	> 0.05
1.2. Men smokers	2.07 (0.95:3.75)	2.09 (0.96:3.76)	> 0.05
2. Women total	0.86 (0.45:1.66)	1.78 (0.78:3.1)	0.001
2.1. Women non-smokers	0.87 (0.48:1.85)	1.56 (0.67:3.49)	0.002
2.2. Women smokers	0.66 (0.39:1.48)	2.01 (0.84:3.02)	0.001

B			
Groups	Healthy people, median value of Ab2 (P25: P75)	Lung cancer patients, median value of Ab2 (P25: P75)	Mann–Whitney U-test for data analysis between groups of healthy people and lung cancer patients, *p* values
1. Men total	6.8 (4.31:10.47)	2.53 (0.9:6.11)	0.001
1.1. Men non-smokers	6.56 (4.21:9.31)	2.22 (1.09:6.13)	0.001
1.2. Men smokers	6.94 (4.63:11.05)	2.53 (0.88:6.36)	0.001
2. Women total	3.26 (1.56:6.08)	2.13 (1:5.52)	> 0.05
2.1. Women non-smokers	3.41 (1.63:6.08)	3.14 (1.15:6.22)	> 0.05
2.2. Women smokers	2.32 (1.35:5.41)	1.76 (0.88:2.92)	> 0.05

C			
Groups	Healthy people, medians value of Ab2/Ab1 (P25: P75)	Lung cancer patients, medians value of Ab2/Ab1 (P25: P75)	Mann–Whitney U-test for data analysis between groups of healthy people and lung cancer patients, *p* values
1. Men total	3.65 (1.86:6.4)	1.35 (0.24:3.8)	0.001
1.1. Men non-smokers	3.58 (1.9:5.92)	1.07 (0.18:2.31)	0.001
1.2. Men smokers	3.87 (1.73:6.4)	1.39 (0.25:3.81)	0.001
2. Women total	3.23 (1.42:8.52)	1.89 (0.34:5.42)	0.001
2.1. Women non-smokers	3.44 (1.53:8.3)	2.03 (0.28:7.52)	0.044
2.2. Women smokers	2.62 (1.42:8.64)	0.99 (0.31:2.52)	0.003

Ab2/Ab1 was obtained by division of the Ab2 level value by Ab1 level value for each examined person (C). Amount of people in each groups in (A), (B), and (C) were the same like in Figure 2A.

The medians values for Ab1 of healthy and lung cancer men groups were about 2 and very similar (Table 1A). The level of Ab1 healthy men was almost two time higher than level of total healthy people on Figure 1A. However, the medians values average for Ab1 healthy women were about 1 and lung cancer women groups were about 2 which completely correlated with Figure 1A. The values of Ab1 levels in healthy women were very different from those in healthy men. Also, the significant differences by Mann–Whitney *U*-test (p = 0.001-0.002) were only between all three groups of healthy and lung cancer women: women total, women non-smokers and women smokers. Men groups did not show that at all. Probably Ab1 level against PAHs was more significant for women then for men. In additives the significant differences were also found only between healthy men and healthy women (*p =* 0.001), between healthy men smokers and healthy women smokers (*p =* 0.001) (data not shown).

The medians values for Ab2 healthy men in all three groups were about 7 (Table 1B). The levels of Ab2 in healthy men were almost 3 times higher than Ab2 levels in lung cancer men groups and also higher than level of total healthy people in Figure 1A. The scatter of median Ab2 values in female groups was larger than in male groups. The median value for Ab2 level of healthy female smokers was the lowest and differed from female non-smokers and the general women group. The median value for Ab2 of non-smokers female with lung cancer was the highest and also differed from those of lung cancer non-smokers and the general lung cancer women groups. The significant differences by Mann–Whitney *U*-test were only found between all three groups of healthy men and lung cancer men (p = 0.001), but not for any women groups. Presumably Ab2 level against PAHs was more important for men then for women. Also, the significant differences were between healthy men and healthy women (*p =* 0.001), healthy men non-smokers and healthy women non-smokers (*p =* 0.001), healthy men smokers and healthy women smokers (*p =* 0.001), lung cancer men smokers and lung cancer women smokers (*p =* 0.02), and lung cancer women non-smokers and lung cancer women smokers (*p =* 0.009) (data not shown).

Table 1C shows the median values of ratio for the Ab2/Ab1, which were obtained by dividing the value of the level Ab2 by the value of the level Ab1 for each person, followed by grouping and calculating the medians for each group. The medians values for Ab2/Ab1 of healthy (~4) and lung cancer men (~1) were in 4 times differed with low spread inside of each groups. These ratios Ab2/Ab1 corresponded to the ratios Ab2/Ab1 of all people (Figure 1B). The medians values for the Ab2/Ab1 of healthy and lung cancer women were more varied probably because of difference in scattering of Ab2 levels in groups of women. For lung cancer women total and lung cancer women non-smokers the Ab2/Ab1 ratio value was about 2, which was higher than the ratio value of medians for the general group of people without grouping (Figure 1B). In contrast the Ab2/Ab1 level value for healthy women smokers was lower than general group of people without grouping. The tendency of Ab2/Ab1 levels values dispersion in groups of healthy and lung cancer women differed from those in men groups. The significant differences by Mann–Whitney *U*-test were between all examined groups of healthy men and women, lung cancer men and women.

So, the analysis of Ab1, Ab2, and Ab2/Ab1 levels in all groups showed significant difference between healthy people and lung cancer patients, also significant difference in immune response in males and females. The ratio of Ab2/Ab1 was more effective in lung cancer prediction rather than Ab levels separately, which correlated with Figure 1.

The smoking factor introduced additional significant differences between the analyzed groups of people by Mann–Whitney *U*-test. For examples, the significant difference was between healthy men smokers and healthy women smokers by Ab1 (*p =* 0.001), healthy men non-smokers and healthy women non-smokers (*p =* 0.001) by Ab2, healthy men smokers and healthy women smokers (*p =* 0.001) by Ab2, lung cancer men smokers and lung cancer women smokers by Ab2 (*p =* 0.02), and lung cancer women non-smokers and lung cancer women smokers by Ab2 (*p =* 0.009). Thus, the smoking factor can affect the levels of Ab1 and Ab2 in human serum when analyzing together with gender and health. However, the smoking factor can be considered as a predictor of lung cancer in terms of Abs levels, but only in combination with a gender predictor, and not independent in the Mann–Whitney *U*-test.

The databases on smokers’ healthy donors and smokers’ lung cancer patients were supplemented with smoking experience and the number of cigarettes smoked per day. A nonparametric Spearman correlation analysis was then carried out in order to find the statistical relationship between random variables: health, Ab1 and Ab2 levels, smoking experience, and smoking intensity. Statistically significant correlations are shown in the Table 2. The ranks coincided with a high probability for the value levels of Ab1 and Ab2; vice versa the value levels of Ab2 and Ab1; the value levels of Ab1 & Ab2 and health; the value levels of Ab1 & Ab2 and smoking experience & smoking intensity. However, the value levels of Ab1 & Ab2 were associated with negative correlation coefficients with smoking parameters. Thus, we can conclude that smoking experience & smoking intensity probably affected on the levels of Ab1 & Ab2 and reduces their concentrations in human serum.

**Table 2:** ELISA data analysis of healthy donors and lung cancer patients’ groups based on Ab1 and Ab2 value levels considering smoking experience and the number of cigarettes smoked per day

	Health status	Smoking experience	Smoking intensity	Ab1	Ab2
Ab1	0.75	–0.3	–0.3		1.0
Ab2	0.75	–0.3	–0.3	1.0	

### Building of lung cancer diagnosis model based by logistic regression and neural networks

The logistic regression was used as a model for prediction of healthy people and lung cancer patients. The age predictor was additionally used in the analysis complementing levels Ab1 and Ab2, gender, and smoking. The age predictor was not specifically used in group analysis by Mann–Whitney *U*-test (Table 1). Because the breakdown of ELISA data by people age with subsequent statistical analysis of the groups gives a distorted version of the effect of such a breakdown on the correlation between groups. That is why we used people age by full rank only in logistic regression calculation. The result of logistic regression calculation without smoking and including smoking predictors is in Table 3.

**Table 3:** The result of logistic regression calculations

	Constant	Abl	Ab2	Gender	Age	Smoking
**Regression**	−6.40	0.06	–0.11	–0.69	0.2	—
**coefficients**	−4.71	0.07	–0.11	–0.14	0.15	–1.69

	Observed	Predicted	Percent
**Non-smoking healthy donors**	133	94	58.04
**Smoking healthy donors**	150	77	65.63
**Non-smoking lung cancer patients**	64	493	91.13
**Smoking lung cancer patients**	67	490	90.57

**Figure 3:**
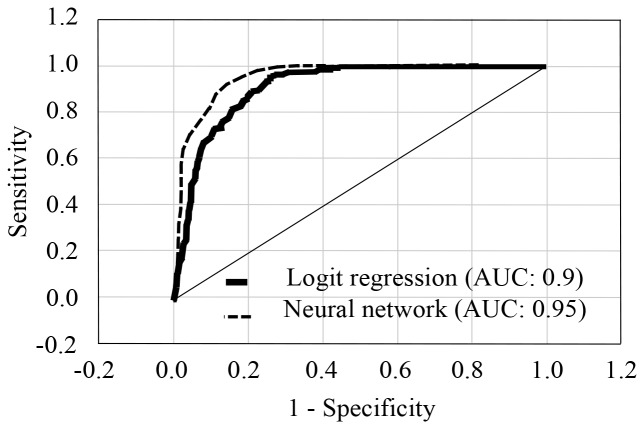
ROC curves for logistic regression and neural networks calculations including smoking predictor.

The sensitivity and specificity for calculations including smoking predictor was 82% and 94%, respectively. The positive predictive was 83.3% and the area under the ROC curve was 0.9 (Figure 3). The sensitivity and specificity for calculations without smoking predictor was 64.9% and 77.5%, respectively. The positive predictive was 81.4% and the area under the ROC curve was 0.81 (data not shown).

It could be seen that smoking affect was the high predictive prognosis (healthy or lung cancer patients) from Table 3, but apparently only in combination with other predictors as gender and age because of features of the logistic regression analysis. The percentage of healthy was 58% and 65.6% for calculations without taking into account and taking into account the smoking predictor, respectively. This confirmed the result obtained by the Mann–Whitney’s analysis of groups (Table 1) and Spearman correlation analysis (Table 2). The smoking predictor had the highest regression coefficient in logistic regression analysis. Others predictors such as levels of Ab1 and Ab2, gender, and age gave almost the same regression coefficients.

Logistic regression model predicted with an accuracy of 58-65.6% for healthy people and with an accuracy of 90.6-91% for lung cancer patients. The evaluation of lung cancer diagnose was overestimated in this model. Used predictors gave well fitted model and will be expected to achieve the same predictive discrimination in a new sample as it appeared to achieve in the developed model.

Finally, the neural networks were done which was statistical method that most closely parallels logistic regression using levels of Ab1 and Ab2, gender, age, and smoking as predictors. The neural networks generated five models using entire human population test above. The best model (70% train sample size, 10% for validation, and 20% test sample size) calculated 0.95 area under the ROC curve with 0.43 cut-off threshold and 93% positive predictive value for lung cancer patients (Figure 3). The neural network model shown the 2.8, 1.82, 1.79, 1.33, and 1.11 sensitivity for smoking, gender, age, Ab1, and Ab2 predictors, correspondingly.

### Prediction of health status using logistic regression model

49 people random sampled from the originally obtained database which we used in analysis of this work and additionally ELISA data for 52 coal miners (only men) were used to evaluate models of logistic regression. Random ELISA data of 49 people contained groups of healthy people and patients with lung cancer as well as all the other groups studied in this paper. The group of 52 coal miners did not include in early calculation and was analyzed by of logistic regression to confirm it predictions. The average of probabilities of health were received and analyzed after Z-conversion as described in Materials and Methods using regression coefficients of logistic regression model with regarding to the smoking factor (Table 3). Table 4 shows the final calculation data.

**Table 4:** The average probability of health status in the groups of random sampled 49 people and 52 coal miners

Groups of people	Healthy	Lung cancer
49 randomly	Mean = 0.29 ± 0.037	Mean = 0.85 ± 0.028
selected people	SD = 0.21-0.38	SD = 0.79-0.9
52 coal miners	Mean = 0.59 ± 0.037	
	SD = 0.52-0.67	

SD was standard deviation.

The logistic regression model worked and proofed data of current manuscript. The model distributed all the analyzed cases between lung cancer patients and healthy people with 80% sensitivity and 85% specificity. The value of average probability for lung cancer patients’ random group (value 0.85) was almost three times higher than for random group of healthy people (value 0.29). It was interesting that the value of average probability of coal miners’ group (value 0.59) was intermediate between healthy donors and lung cancer patients. It means that coal miners probably belonged to group of cancer risk.

**Figure 4:**
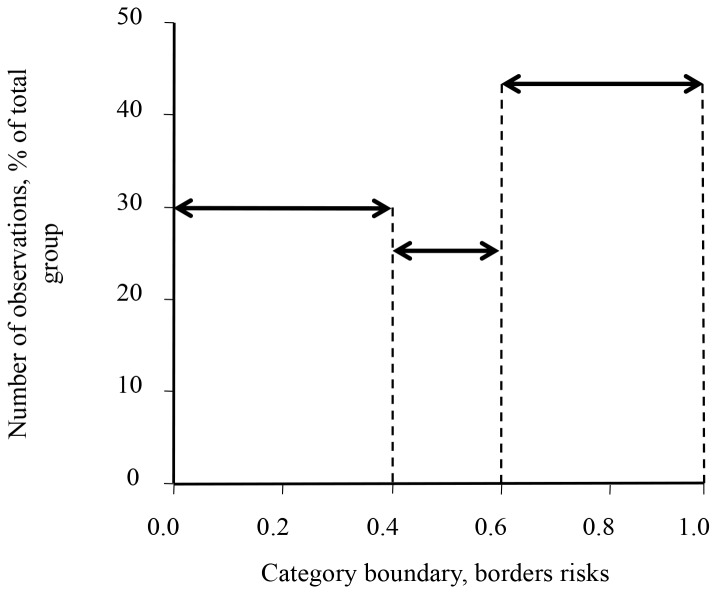
Borders of lung cancer risks in examined group of coal miners. The borders of cancer risk prediction were from 0.0 as a negative symptom for healthy people till 1.0 as a positive symptom for lung cancer patients.

Than the all values of probabilities of 52 coal miners were placed on a scale from 0 till 1 (Figure 4). Value 0.0 was supposedly accepted for healthy people (absence of disease) and value 1.0 for lung cancer patients (presence of disease).

According to this analysis, the group of coal miners was divided into three subgroups: 16 coal miners with low risk of lung cancer (value of probability 0-0.4), 13 coal miners with middle risk of lung cancer (value of probability 0.4-0.6), and 23 coal miners with high risk of lung cancer (value of probability 0.6-1) in the examined group. Also, it was seen from individual analysis data of each coal miner that lung cancer risk in this population depended from age. For example, nonsmoking 62 years old coal miner had 0.95 probability of lung cancer risk or nonsmoking 54 years old coal miner had 0.8 probability of lung cancer risk compared to the nonsmoking 38 years old coal miner with 0.34 probability of lung cancer risk or nonsmoking 43 years old coal miner with 0.41 probability of lung cancer risk (data not shown). Definitely this study needs analyzes more blood serums samples of coal miners for further investigation.

Thus, our proposed new biomarkers can be used to predict risk of lung cancer, since the ratio of idiotypic and anti-idiotypic antibodies against polycyclic aromatic hydrocarbons levels in human blood serum are higher in healthy people than in patients with lung cancer.

## DISCUSSION

In these prospective data, we observed that the levels of Ab1 and Ab2 against PAHs in human blood serum diffed in lung cancer patients and healthy donors. The ratio of Ab2/Ab1 was more evident marker for that. The present research was confirmed our previous data where the production of Ab1 and Ab2 against Bp in non-immunized mice was similar to those in healthy persons and the increasing levels the same Abs in immunized mice possessed similar patterns to that in lung cancer patients [22]. The Ab2/Ab1 ratios were 3.95 (1.84:7.51) and 1.19 (0.52:2.62) for healthy people and lung cancer patients, correspondingly. We could speculate about low ratio of Ab2/Ab1 in blood serum of lung cancer patients compared with healthy persons, because of changing the whole immune status in lung cancer.

The breakdown into groups of patients with lung cancer and healthy people according to gender and smoking showed that in these groups there were enough cases for further calculations (Figure 2A). The analysis of lung cancer stages, type of lung cancer, chemotherapy, and surgery in the group of patients with lung cancer (Figure 2B) shown absent correlations and probably did not affect the levels of Ab1 and Ab2 in human blood serum. However, the analysis of the ELISA data of healthy donors and lung cancer patients grouped and broken down with gender and smoking revealed that the median for Ab1 levels in patients and healthy individuals differ only in women but not in men (Table 1A). Moreover, the smoking factor in women influenced the level of Ab1. The situation was the opposite in the case of Ab2 levels, which appear to be more significant for men but not for women (Table 1B). However, the difference between healthy and lung cancer patients in all groups was obvious in the case of the analysis of the Ab2/Ab1 ratio. In the last case, the smoking factor influenced the levels of Ab2/Ab1. Thus, first, the immune response in men was different from that in women. Second, the measurement of the Ab2/Ab1 ratio gave a clearer result for the difference between healthy donors and lung cancer patients. Third, smoking as a risk factor alone did not affect Ab1 and Ab2 levels, but only in combination with other factors such as gender. These findings confirm our previous results where we found that smoking did not affect Ab1 and Ab2 levels in lung cancer patients and healthy individuals in small number of examined samples but without any breakdown into groups [22]. The relationship between cancer and cigarette smoking has been well established in many types of cancers [42]. In current manuscript additional analysis ELISA of healthy donors and lung cancer patients’ data broken down according to smoking experience and the number of cigarettes smoked per day shown statistically significant correlations with negative correlation coefficients. Probably, that was meaning that the experience of smoking and the smoking intensity reduces concentrations of Ab1 and Ab2 against PAHs in human serum.

In the logistic regression calculations and neural networks, we applied an additional age factor as a risk factor for lung cancer. This was done in order not to divide the group of lung cancer patients and healthy people into discrete age groups, but to analyze all available age values at once. We also did two logistic regression analyses separately: without and with the smoking factor (Table 3). The calculations have shown that age gave additional importance and weight of predicting lung cancer, as well as smoking. To confirm prediction of lung cancer by model of logistic regression the random ELISA data of 49 people from already analyzed data (healthy donors and lung cancer patients) and the group of 52 coal miners (only men; the new group did not include in calculation above) were calculated by a model that divided all people into lung cancer patients and healthy people, which coincided with the available information on individuals (Table 4). The value of average probability of coal miners was in between the value of average probability of healthy and the lung cancer patients. All the coal miners who participated in this study did not have lung cancer. Therefore, we concluded that the coal miners belong to the so-called risk of lung cancer group. It is confirmed already published data that coal miners belonged to risk group of lung cancer [43, 44]. That is why coal miners can have increased concentrations of Abs against PAHs in blood serum. We have broken down the coal miners’ data of values probabilities got from the logistic regression model test into three groups of coal miners with varying degrees of risk for lung cancer: low, medium, and high (Figure 4). It is interesting that the degree of risk of coal miners directly depended on the age of the individual. Definitely, this requires further work and analysis of large blood serum samples of coal miners. So, the results of current manuscript could involve the formation of risk groups and preventive examination of people.

The influence of individual factors including industrial and domestic factors on the levels and ratio of Ab1 and Ab2 would be the next subject of the research. Also, it would be interesting to figure out if Ab1/Ab2 ratio depends on the activity of chemical carcinogens metabolizing enzymes and levels of Abs against estradiol and progesterone in human blood serum (both men and women).

## MATERIALS AND METHODS

### Clinical material

The blood serums of 784 people, including 557 lung cancer patients and 227 healthy people without cancer, were obtained from the Regional Clinical Oncology Center in Kemerovo, Russia and Kemerovo blood transfusion station, Russia, respectively. The peripheral blood was carried out with the informed consent of the people. Details of a patients age, gender, out-come, date of diagnosis and disease substratification were restricted by the agreement with the donating clinics.

### Mouse Ab1 scFvs against PAHs purification

pSh was mouse Ab1 scFvs against PAHs. The pSh purification was processed as described earlier [39]. cDNA was cloned into plasmid pTT10 (kindly provided to us Institute of Chemical Biology and Fundamental Medicine Siberian Branch of the Russian Academy of Sciences, Novosibirsk) on the restriction sites NcoI and BamHI. The final DNA construct contained the cDNA of scFv and a fusion protein, cellulose binding domain (CBD). The final protein was named pSh-CBD. The *E.coli* strain M15 was used for pSh-CBD expression. Induction of protein synthesis was carried out by addition of isopropylthio-β-galactoside (IPTG) to a final concentration of 1 mM. The bacterial cells were collected by centrifugation after 4 hr of incubation at +37° C. Disrupting the bacterial cells walls and the chromosomal DNA was by ultrasonic homogenizer Sonics. Synthesized pSh-CBD was mainly located in the insoluble cell fraction. It was separated by centrifugation. The precipitate was homogenized in 8 M urea. Adsorption of the protein was carried out on amorphous cellulose with 2 M urea. pSh-CBD was eluted from the cellulose with 4 M guanidine hydrochloride followed by dialysis against 400 mM Tris, 500 mM NaCl, 1 mM EDTA buffer solution for 4 hr. Protein expression and the degree of purification were assessed by electrophoresis in 12% polyacrylamide gel according to the method of Laemmli.

The specificity of pSh binding was in details described in [39]. The pSh bound to group of PAHs with high affinity: *K_d_* values of pSh for Bp – 0.705 × 10^−8^ M, chrysene – 3.23 × 10^−8^ M, pyrene – 2.73 × 10^−7^ M, anthracene – 6.49 × 10^−8^ M, benz[a]anthracene – 2.36 × 10^−8^ M, and estradiol as negative control – 1.68 × 10^−4^ M.

### Synthesis of conjugates

Conjugate Bp-BSA was synthesized by covalent coupling of hapten aldehyde group to the BSA amino groups [40]. The 1.5 ml pyridine was added drop by drop with stirring and cooling in 0.1 g BSA in 1 ml of 0.1 N NaOH follow adding 16 mg of benzo[a]pyrene-6-carboxaldehyde. After three hours of stirring at 25° C, a blocking compound of 50 mg of acrylamide and a solution of 10 mg of sodium borohydride reducing agent in 1 ml of water were added. After 30 minutes, 4 drops of glacial acetic acid were added and conjugate was precipitated by 8 ml of acetone. After 15 minutes, the precipitate was centrifuged and washed 5 times by 8 ml of acetone. After drying under vacuum the conjugate was dissolved in 10 ml of 0.01 N NaOH. The conjugate was stored in a soluble form in pH 7.2-7.4. Insoluble polymer products were not formed when the solution was stored at a temperature of +5° C for at least six months.

### ELISA

The checking of the Ab1 and Ab2 against PAHs in human blood serum was by noncompetitive ELISA [41]. The immunological polystyrene plates were coated with 50 μl of 2 μg/ml Bp-BSA at 25° C overnight or 50 μl of pSh-CBD at a concentration of 50 ng/μl for 1 h at 37° C. The control wells were incubated with 50 μl 0.5% BSA or 50 μl of 50 μg/ml CBD. Then the plates were blocked by 100 μl of PBS containing 0.5% BSA and 0.05% Tween 20 for 1 h at 25° C with shaking.. The human serums were diluted by blocking solution for the analysis at 1:100. The plates were coated by diluted human serums and incubated at 37° C for 1 h on a shaker. After this step the plates were washed 3 times with 100 μl of PBS/0.05% Tween 20. The Ab1/Ab2 binding to the wells was introduced into 50 μl of HRP labeled anti-human immunoglobulin G at 37° C on a shaker for 1 h. Absorbance was performed using tetramethylbenzidine at 450 nm.

Levels of Ab1 against Bp in human blood serum were calculated using formula: Ab1 = (OD Bp-BSA – OD BSA) / OD BSA. The OD Bp-BSA and OD BSA were optical densities with adsorbed Bp-BSA and BSA, respectively. Levels of Ab2 against PAHs in human blood serum were calculated using formula: Ab2 = (OD pSh-CBD – OD CBD)/OD CBD. The OD pSh-CBD and OD CBD were optical densities with adsorbed pSh-CBD and CBD, respectively.

### Statistical analysis

Excel 2010 and Statistica 10 were applied in the analysis of data and calculations. Values p<0.05 were considered statistically significant. The methods and criteria of modified Shapiro-Wilk were used to assess the normality distribution of the ELISA data. Ejection points were more than three standard deviations from the median removed by box-plots analysis. Amount of cases in each group was done by analysis of Pearson’s chi-squared test. The final regrouped data were analyzed by Z adjusted Mann–Whitney *U*-test. Correlations between groups were calculated by Spearman correlation analysis. The logistic regression and neural networks was used as a prognostic model for the diagnosis of lung cancer. The received regression coefficients of logistic regression with regard to the smoking factor and without were applied. The value of average probabilities for healthy donors/lung cancer patients were received and analyzed after Z-conversion using formula:

$$\begin{array}{l} \text{1}/\text{(1}+{\text{e}}^{\text{-z}}\text{),}\;\text{z}=\text{(constant}\;\text{of}\;\text{regression}\;\text{coefficient}\\ \;\;\;\;\;\;\;\;\;\;\;\;\;\;\;\;\;\;\;\;\;\;\;\;\;\;\;\;\;\;\;+\;\text{Ab1}\;\text{regression}\;\text{coefficient}\times \text{Ab1}\;\text{level}\\ \;\;\;\;\;\;\;\;\;\;\;\;\;\;\;\;\;\;\;\;\;\;\;\;\;\;\;\;\;\;\;+\text{Ab2}\;\text{regression}\;\text{coefficient}\;\times \text{Ab2}\;\text{level}\\ \;\;\;\;\;\;\;\;\;\;\;\;\;\;\;\;\;\;\;\;\;\;\;\;\;\;\;\;\;\;\;+gender\;\text{regression}\;\text{coefficient}\times \text{gender}\\ \;\;\;\;\;\;\;\;\;\;\;\;\;\;\;\;\;\;\;\;\;\;\;\;\;\;\;\;\;\;\;+\text{age}\;\text{regression}\;\text{coefficient}\times \text{age}+\text{smoking}\;\\ \;\;\;\;\;\;\;\;\;\;\;\;\;\;\;\;\;\;\;\;\;\;\;\;\;\;\;\;\;\;\;\;\;\;\;\text{regression}\;\text{coefficient}\times \text{smoking)} \end{array}$$

The risks of lung cancer in coal miners were also analyzed after Z-conversion of ELISA data taking into account regression coefficients of logistic regression model with regarding to the smoking factor. Received the value of averages probability of coal miners were distributed between 0.0 (supposedly accepted for healthy donors) and 1.0 (for lung cancer patients).
